# Propentofylline Prevents Sickness Behavior and Depressive-Like Behavior Induced by Lipopolysaccharide in Rats via Neuroinflammatory Pathway

**DOI:** 10.1371/journal.pone.0169446

**Published:** 2017-01-05

**Authors:** Márcia M. T. Moraes, Marcella C. Galvão, Danilo Cabral, Cideli P. Coelho, Nicolle Queiroz-Hazarbassanov, Maria F. M. Martins, Eduardo F. Bondan, Maria M. Bernardi, Thiago Berti Kirsten

**Affiliations:** 1 Environmental and Experimental Pathology, Paulista University, Sao Paulo, Brazil; 2 Department of Pathology, School of Veterinary Medicine, University of São Paulo, Sao Paulo, Brazil; 3 Graduate Program of Animal Medicine and Welfare, University of Santo Amaro, Sao Paulo, Brazil; Radboud University Medical Centre, NETHERLANDS

## Abstract

Recent studies have demonstrated the intimate relationship between depression and immune disturbances. Aware of the efficacy limits of existing antidepressant drugs and the potential anti-inflammatory properties of propentofylline, we sought to evaluate the use of propentofylline as a depression treatment. We used a rat model of depression induced by repetitive lipopolysaccharide (LPS) administrations. We have studied sickness behavior, by assessing daily body weight, open field behavior, and TNF-α plasmatic levels. Anxiety-like behavior (light-dark test), depressive-like behavior (forced swim test), plasmatic levels of the brain-derived neurotrophic factor (BDNF, depression biomarker), and central glial fibrillary acidic protein (GFAP) expression (an astrocyte biomarker) were also evaluated. LPS induced body weight loss, open field behavior impairments (decreased locomotion and rearing, and increased immobility), and increased TNF-α levels in rats, compared with control group. Thus, LPS induced sickness behavior. LPS also increased the immobility and reduced climbing in the forced swim test, when compared with the control group, i.e., LPS induced depressive-like behavior in rats. Propentofylline prevented sickness behavior after four days of consecutive treatment, as well as prevented the depressive-like behavior after five days of consecutive treatments. Propentofylline also prevented the increase in GFAP expression induced by LPS. Neither LPS nor propentofylline has influenced the anxiety and BDNF levels of rats. In conclusion, repetitive LPS administrations induced sickness behavior and depressive-like behavior in rats. Propentofylline prevented both sickness behavior and depressive-like behavior via neuroinflammatory pathway. The present findings may contribute to a better understanding and treatment of depression and associated diseases.

## Introduction

Depression is a complex mood disorder, characterized by loss of interest or pleasure, anhedonia, apathy, poor concentration, low energy, disturbed sleep and appetite, reduced social and sexual interest, among other symptoms [1, 2]. It is estimated that 40 to 60% of suicides are directly linked to depression [3, 4]. Over 15% of all adults will experience at least one episode of major depression at some point in their lifetime, being women more affected than men (20% vs. 10%) [5, 6]. The costs related to this disorder represent an economic burden of tens of billions of dollars per year [7]. Therefore, depression has been considered as the disease of this century.

Unfortunately, little is still known about the etiology and pathophysiology of depression. It is regarded as a disorder of multifactorial causes, including genetic factors, stressful events, diseases, hormonal imbalance, and drug abuse [2, 8]. Although the monoaminergic (serotonin and noradrenaline) hypothesis is well recognized and accepted, and is also the basis for supporting antidepressants prescription, it fails to explain and treat many aspects of depression [9]. Smith [10] proposed the macrophage theory of depression, which states that the excessive secretion of interleukin (IL)-1 and other products of macrophages are involved in the pathogenesis of depression. In this sense,https://translate.google.com/?tr=t&hl=pt-BR some patients diagnosed with depression have increased levels of cytokines such as tumor necrosis factor (TNF-α) and IL-6 in the blood [11]. Hepatitis C or cancer patients treated with interferon alpha (IFN-α) also developed depression [12]. Moreover, even low doses of lipopolysaccharide (LPS) administered to volunteer subjects are able to increase serum levels of proinflammatory cytokines and induce anhedonia, which is one of the main symptoms of depression [13]. LPS is an endotoxin that mimics infection by gram-negative bacteria by activating the immune system to release cytokines, such as TNF-α, IL-1β, and IL-6 [14–16].

Based on these neuroimmune aspects, many drugs have been tested for the treatment of depression, especially the use of anti-inflammatory drugs [9]. For example, the cyclooxygenase-2 inhibitor celecoxib, that inhibits proinflammatory cytokines production, has therapeutic effects in depressive patients treated with reboxetine [17]. Similar results were found with the association of celecoxib with fluoxetine [18]. TNF-α inhibitors, such as etanercept and infliximab reduce depressive symptoms in patients with psoriasis and Crohn’s disease and have been examined as potential treatments for depressive patients [19, 20]. In this sense, due to the potential anti-inflammatory characteristics of propentofylline, we proposed it as a candidate for depression treatment. Propentofylline (3-methyl-1-(5’-oxohexyl)-7-propylxanthine), a xanthine derivative, presents strong neuroprotective, antioxidant and some anti-inflammatory effects [21, 22]. Clinically, it has shown efficacy in degenerative vascular dementia and as a potential adjuvant treatment to Alzheimer’s disease, schizophrenia, and multiple sclerosis [21]. Propentofylline acts as a glial modulator and inhibits macrophagic TNF-α production [23].

Because of the efficacy limitation of existing antidepressant drugs, the objective of this study was to test propentofylline as a potential antidepressive-like effect inductor evaluated in the forced swim test. We used a rat model of depressive-like behavior induced by repetitive LPS administrations [24–27]. First, we evaluated sickness behavior induction and remission based on the model described by Dantzer et al. [26], evaluating daily body weight, daily open field behavior, and TNF-α plasmatic levels. Anxiety-like behavior was evaluated with the light-dark test. Depressive-like behavior was evaluated with the forced swim test. Besides TNF-α [11], the brain-derived neurotrophic factor (BDNF) has also been considered as a depression biomarker [28, 29] and its plasmatic levels were evaluated. Lastly, expression of glial fibrillary acidic protein (GFAP), which is an astroglial pathology biomarker in neurological diseases [30], was evaluated in the medial prefrontal cortex, nucleus accumbens, and hippocampus.

## Materials and Methods

### Ethics statement

The present study was carried out in strict accordance with the recommendations of the Guide for the Care and Use of Laboratory Animals of the National Institutes of Health [31]. The protocol was approved by the Committee on the Ethics of Animal Experiments of the Paulista University, Brazil (Permit Number: 296/14). All efforts were made to minimize suffering, reduce the number of animals used, and utilize alternatives to *in vivo* techniques when available. The experiments were also performed in accordance with good laboratory practice protocols and quality assurance methods.

### Animals

A total of 40 Wistar male rats (*Rattus norvegicus*) with 95–115 days of age and weighing 305–375 g from the School of Veterinary Medicine (University of Sao Paulo, Sao Paulo, Brazil) were used. They were housed in polypropylene cages (45.5 X 34.5 X 20 cm; 5 rats per cage) with microisolator system (Tecniplast, Buguggiate, Italy), controlled temperature (22°C ± 2°C) and humidity (55–65%) with artificial lighting (12-hr light/12-hr dark cycle, lights on at 7:00 AM). The animals had free access to irradiated rodent chow (BioBase, Águas Frias, Brazil) and filtered water. Sterilized and residue-free wood shavings were used for animal bedding.

### Treatments and groups

Rats were treated with propentofylline solution and/or LPS solution and/or their vehicle, as described below. Propentofylline was administered at 12.5 mg/kg/day single dose (Agener União Química, Sao Paulo, Brazil, 20 mg/mL solution) by intraperitoneal (i.p.) route [22]. Rats received propentofylline for five consecutive days. LPS (from *Escherichia coli*; Sigma, St. Louis, USA, serotype 0127: B8) was dissolved in sterile saline (1 mg/mL LPS in a 0.9% NaCl solution) and administered i.p. at a dose of 1 mg/kg/day, based on Bay-Richter et al. [24] studies. This dose is considered able to induce sickness behavior for at least 24 hours, without sepsis [24]. Rats received LPS for two consecutive days, on days 3 and 4 of propentofylline treatment. Sterile saline solution (0.9% NaCl) was administered as vehicle/control groups. Each rat schedule with saline treatment received a 0.1 mL/100 g, i.p., of saline solution.

The rats were randomly divided into four groups (n = 10 per group). (1) SAL+SAL (control group), rats that received saline solution for five consecutive days. On days 3 and 4 they also received an additional saline dose 1 hour after the first injection. (2) SAL+LPS (LPS group), rats that received saline solution for five consecutive days. On days 3 and 4 they also received a LPS dose 1 hour after the saline injection. (3) PPF+LPS, rats that received propentofylline solution for five consecutive days. On days 3 and 4 they also received a LPS dose 1 hour after the propentofylline injection. (4) PPF+SAL (propentofylline group), rats that received propentofylline solution for five consecutive days. On days 3 and 4 they also received a saline dose 1 hour after the propentofylline injection.

### Sickness behavior

Sickness behavior is normally a temporary state characterized by adaptive behavioral- and neuroimmune-specific changes orchestrated by the host to fight the invading microorganism and heal more quickly, as well as to reduce exposure of the sick animal to predation and contamination of their colony [32, 33]. Some of the most typical symptoms of the sickness behavior are prostration, decreases in exploratory activity and in feeding behavior, weight loss, and increase of peripheral proinflammatory cytokines levels (such as TNF-α) [34, 35]. Thus, we evaluated the open field general activity and body weight of rats daily, as well as the plasmatic TNF-α levels.

Body weight (g) was evaluated daily throughout the five days of treatment. Open field behavior was evaluated three times, 1 hour after the LPS/vehicle injections (days 3 and 4) and 24 hours after the last LPS/vehicle injection (day 5). The open-field apparatus is used to evaluate exploratory/motor behaviors [36]. It consisted of a round wooden arena (96 cm diameter, 29 cm high walls) that was painted gray with an acrylic washable cover and subdivided into 25 parts. Each rat was individually placed in the center of the apparatus, and the following parameters were evaluated over a period of 5 min: locomotion frequency (number of floor units entered with all four paws), rearing frequency (number of times the rodents stood on their hind legs), and total immobility time (s). The testing room, which was isolated from the experimenter, was a small room with dim lighting. A video camera mounted above the arena was used to collect the data. The apparatus was washed with a 5% alcohol/water solution before placement of the animals to obviate possible biasing effects from odor cues left by a previous rat.

### Anxiety-like behavior

Immediately after the last open field test (day 5), rats were observed in a light-dark apparatus to evaluate anxiety-like behavior [37]. This model is based on the innate aversion of rodents to bright places, generating an inherent conflict between their exploratory drive to a novel place and their avoidance of the lit compartment [37, 38]. The apparatus consisted of an acrylic box (80 cm length, 40 cm width, 30 cm high) containing two compartments (separated by a door with 13 x 8 cm): dark room with black walls and floor (34 cm length), and light room, with white walls and floor (44 cm length) and illuminated with white fluorescent lamp (15W, 4100K). Each rat was individually placed in the center of the light room, facing the wall opposite to the door. The following parameters were evaluated over a period of 5 min: dark side entry latency (s), total time (s) spent in the dark side, total time (s) spent in the light side, and rearing frequency. The testing room, which was isolated from experimenter, was a small room with dim lighting. A video camera mounted above the arena was used to collect the data. The apparatus was washed with a 5% alcohol/water solution before placement of the animals to obviate possible biasing effects from odor cues left by previous rat.

### Depressive-like behavior

Immediately after the light-dark test (day 5), rats were observed in the forced swim test to evaluate depressive-like behavior. This test is the most widely used tool for assessing antidepressant activity preclinically [39]. It is based on the observation that rats, following initial escape-oriented movements, develop an immobile posture when placed in an inescapable cylinder of water. The immobility is thought to reflect a failure of persistence in escape-directed behavior (i.e., behavioral despair) [39]. In this model, the longer the rats remain immobile and not trying to escape (such as climbing), the more they are considered to exhibit depressive-like behavior. The apparatus consisted of a round transparent acrylic arena (46 cm height, 20 cm diameter) containing 30 cm water at 23°C ± 1°C. Each rat was individually and gently placed on the water surface, and the following parameters were evaluated over a period of 7 min: first immobility latency (s), total immobility (s), and total time (s) spent climbing. Immobility was considered the absence of active behavior, i.e., when the rat was not swimming or climbing, remaining passively floating, or performing only minimal movements necessary to keep the nose above the water. The water in the cylinder was changed after each animal observation to avoid olfactory cues left by the previous rat.

### Plasmatic evaluations

Immediately after the forced swim test (day 5), rats were decapitated and the trunk blood was collected in conical tubes that contained 10% ethylenediaminetetraacetic acid (EDTA). The samples were centrifuged (3.500 RPM, 15 min, 15°C), and plasma was obtained. Plasma samples of each animal were aliquoted and stored in different microtubes for separate analyses of TNF-α and BDNF using enzyme-linked immunosorbent (ELISA) commercial kits in duplicate and according to the manufacturer’s instructions.

TNF-α was quantified using the DuoSet R&D Systems kit (cat. no. DY510, Minneapolis, USA). TNF-α is considered a biomarker of sickness behavior [35, 40] and depression [11]. BDNF levels were determined using a Promega kit (cat. no. G7610, Madison, USA). BDNF has also been considered as a depression biomarker [28, 29]. In both cases, the results are expressed in pg/ml.

### Astrocyte GFAP immunohistochemistry

Simultaneously with the blood collection, the brains of the rats were collected and fixed in 10% buffered formalin for 48 h. Coronal sections of each brain were made to reach the medial prefrontal cortex, the nucleus accumbens, and the hippocampus. These brain areas are involved in the pathophysiology of depression, with studies of both depressive patients and mice models of depression (unpredictable chronic mild stress) [41, 42]. Incidentally, it has been postulated that dysfunctions of glial cells, especially astrocytes, play a critical role in the pathogenesis of depression [42]. GFAP immunohistochemical procedure using the avidin-biotin peroxidase complex (ABC) method was performed as described previously [43]. We used polyclonal rabbit anti-GFAP immunoglobulin (1:1000; Z0334, Dako, Glostrup, Denmark) as the primary antibody and biotinylated secondary antibody (K0690, Dako Universal LSAB 2 System, HRP, Glostrup, Denmark). Eight photomicrographs from each individual prefrontal cortex and hippocampus section, and four from each nucleus accumbens section were made using a 40x objective. The area of astrocytes and their processes, marked in brown, was automatically calculated, in pixels, using Metamorph software (Molecular Devices, Sunnyvale, USA) calibrated with digital color filters that regulated red, green, and blue bits such that only positive cells were included and background staining was excluded from the measurement.

### Statistical analysis

Homogeneity and normality was verified using a Bartlett’s test. One-way analysis of variance (ANOVA) followed by Newman-Keuls’s multiple comparison test was used to compare the parametric data among the four groups. For analysis that includes evaluations in consecutive days, two-way ANOVA followed by Newman-Keuls’s multiple comparison test was used. The results are expressed as the mean ± SEM. In all cases, the results were considered significant if *p* < 0.05.

## Results

**Fig 1:**
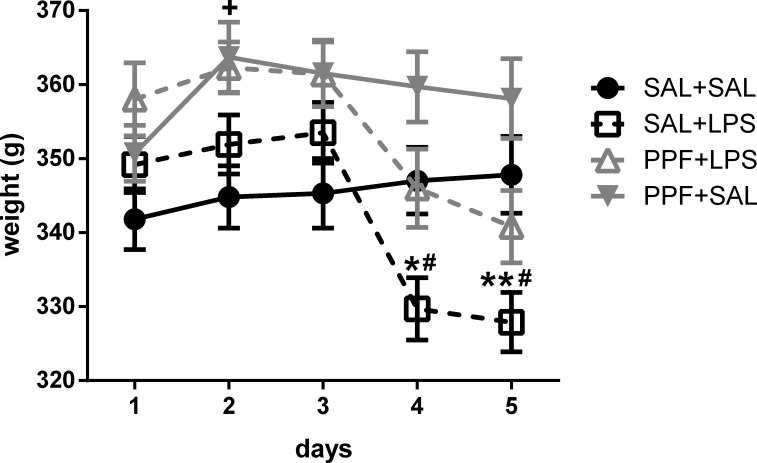
Body weight. Effects of LPS (1 mg/kg/day) and propentofylline (12.5 mg/kg/day) on the body weight of adult male rats. SAL+SAL, saline injection at days 1–5 and another saline injection 1 h later at days 3–4; SAL+LPS, saline injection at days 1–5 and LPS injection 1 h later at days 3–4; PPF+LPS, propentofylline injection at days 1–5 and LPS injection 1 h later at days 3–4; PPF+SAL, propentofylline injection at days 1–5 and saline injection 1 h later at days 3–4 (*n* = 10 per group). *p < 0.05 and **p < 0.01, SAL+LPS vs. SAL+SAL; ^#^*p* < 0.05, SAL+LPS vs. PPF+LPS; ^+^*p* < 0.05, SAL+SAL vs. PPF+SAL (two-way ANOVA followed by the Newman-Keuls test). The data are expressed as the mean ± SEM.

As shown in S1 Table, we found significant effect for treatment, days, and interaction factors for the body weight analysis. LPS (SAL+LPS group) reduced the body weight in the second day of administration and 24 hours after the last LPS administration, compared with the control (SAL+SAL) group, i.e., at days 4 and 5 of the experiment (Fig 1). Propentofylline treatment together with LPS (PPF+LPS group) prevented the body weight loss induced by LPS both at days 4 and 5, compared with SAL+LPS group, reaching the same levels exhibited by the control group. Propentofylline alone (PPF+SAL group) increased the body weight compared with the control group only at day 2.

**Fig 2:**
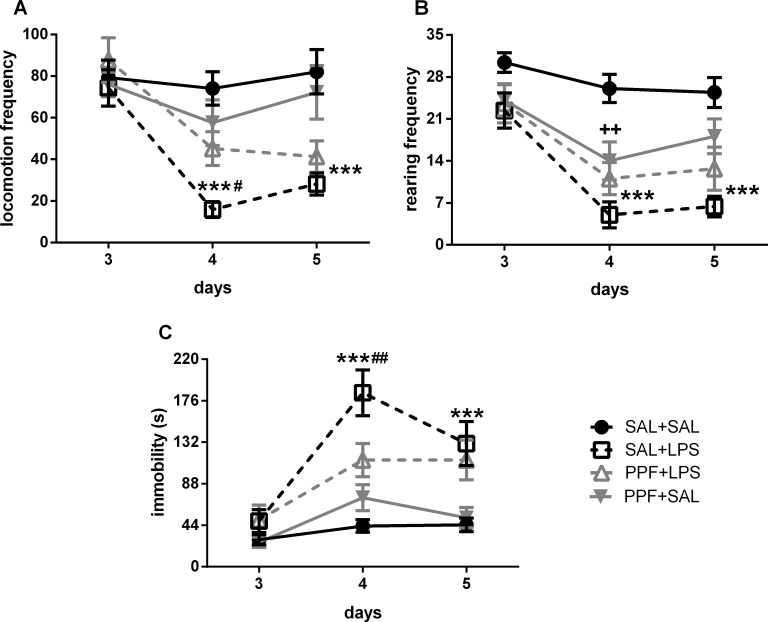
Open-field behavior. Effects of LPS (1 mg/kg/day) and propentofylline (12.5 mg/kg/day) on the open-field behaviors in adult male rats. SAL+SAL, saline injection at days 1–5 and another saline injection 1 h later at days 3–4; SAL+LPS, saline injection at days 1–5 and LPS injection 1 h later at days 3–4; PPF+LPS, propentofylline injection at days 1–5 and LPS injection 1 h later at days 3–4; PPF+SAL, propentofylline injection at days 1–5 and saline injection 1 h later at days 3–4 (*n* = 10 per group). ***p < 0.001, SAL+LPS vs. SAL+SAL; ^#^*p* < 0.05 and ^##^*p* < 0.01, SAL+LPS vs. PPF+LPS; ^++^*p* < 0.01, SAL+SAL vs. PPF+SAL (two-way ANOVA followed by the Newman-Keuls test). The data are expressed as the mean ± SEM.

S1 Table also shows a significant effect for treatment, days, and interaction factors for the locomotion and immobility analysis in the open field. For the rearing parameter, we found a significant effect only for treatment and days factors. LPS (SAL+LPS group) reduced the locomotion and rearing frequencies and increased immobility time in the second day of administration and 24 hours after the last LPS administration, compared with the control group, i.e., at days 4 and 5 of the experiment (Fig 2). Propentofylline treatment together with LPS (PPF+LPS group) prevented the locomotion reduction and the immobility increase induced by LPS at day 4, compared with SAL+LPS group, reaching the same levels exhibited by the control group. Although there was a strong effect for propentofylline when together with LPS in the open field, preventing the behavioral impairments induced by LPS, propentofylline alone (PPF+SAL group) resulted only in a slight behavioral effect. PPF+SAL decreased the rearing frequency compared with the control group only at day 4, not changing locomotion and immobility parameters.

**Fig 3:**
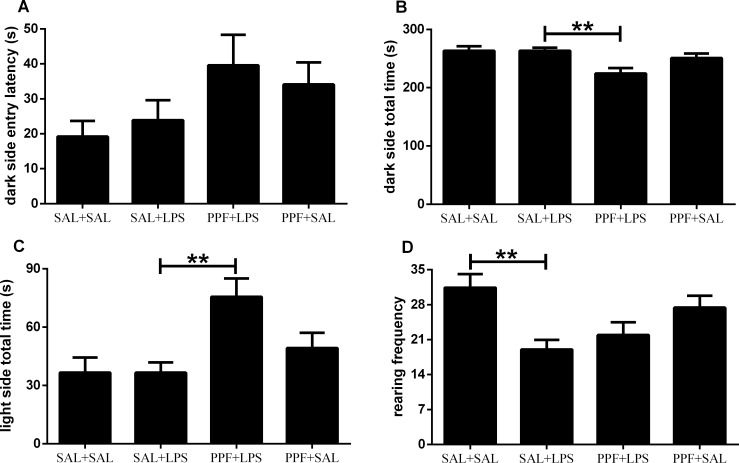
Anxiety-like behavior. Effects of LPS (1 mg/kg/day) and propentofylline (12.5 mg/kg/day) on the light-dark test in adult male rats. SAL+SAL, saline injection at days 1–5 and another saline injection 1 h later at days 3–4; SAL+LPS, saline injection at days 1–5 and LPS injection 1 h later at days 3–4; PPF+LPS, propentofylline injection at days 1–5 and LPS injection 1 h later at days 3–4; PPF+SAL, propentofylline injection at days 1–5 and saline injection 1 h later at days 3–4 (*n* = 10 per group). **p < 0.01 (one-way ANOVA followed by the Newman-Keuls test). The data are expressed as the mean ± SEM.

The light-dark performance was different between groups for both the dark and light side total times, and the rearing frequency, but not for the dark side entry latency (S2 Table). Propentofylline treatment together with LPS (PPF+LPS group) decreased the time spent in the dark side, increasing the time spent in the light side, compared with the LPS (SAL+LPS) group (Fig 3). Although we observed an effect for propentofylline when together with LPS, compared with SAL+LPS group in the light-dark test, LPS or propentofylline alone (SAL+LPS and PPF+SAL groups) did not influence the anxiety-like parameters, compared with control group. When analyzing the motor/exploratory parameter in the light-dark test, we found a decrease in rearing frequency induced by LPS (SAL+LPS) group, compared with control group. This motor/exploratory impairment induced by LPS in the light-dark test was the same as found in the open-field test.

**Fig 4:**
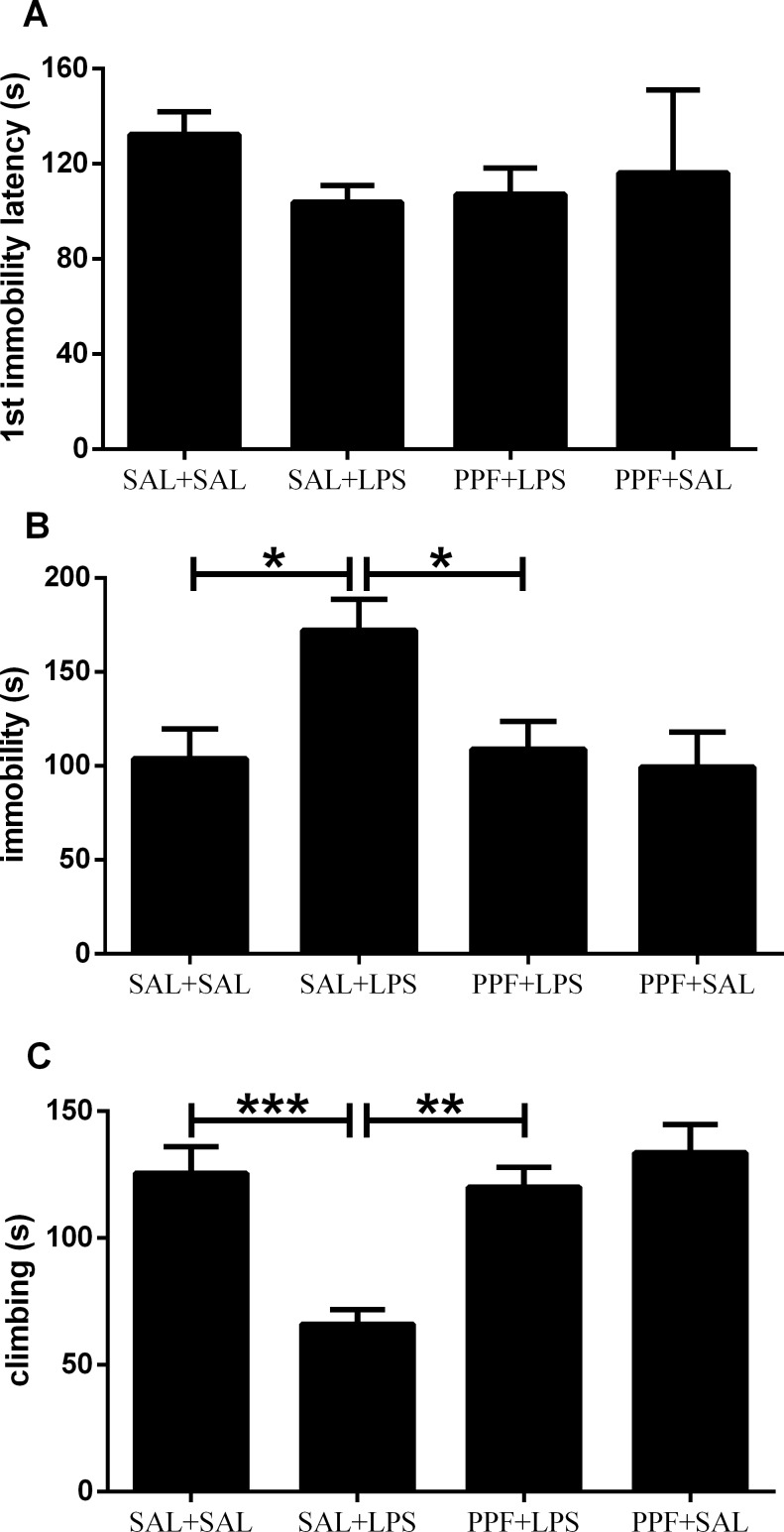
Depressive-like behavior. Effects of LPS (1 mg/kg/day) and propentofylline (12.5 mg/kg/day) on the forced-swim test in adult male rats. SAL+SAL, saline injection at days 1–5 and another saline injection 1 h later at days 3–4; SAL+LPS, saline injection at days 1–5 and LPS injection 1 h later at days 3–4; PPF+LPS, propentofylline injection at days 1–5 and LPS injection 1 h later at days 3–4; PPF+SAL, propentofylline injection at days 1–5 and saline injection 1 h later at days 3–4 (*n* = 10 per group). *p < 0.05, **p < 0.01, ***p < 0.001 (one-way ANOVA followed by the Newman-Keuls test). The data are expressed as the mean ± SEM.

The forced-swim performance was different between groups for the immobility and total climbing time, but not for the first immobility latency (S2 Table). LPS (SAL+LPS group) increased the immobility time and reduced the climbing time, compared with the control group (Fig 4). Propentofylline treatment together with LPS (PPF+LPS group) prevented the immobility and climbing impairments induced by LPS, compared with SAL+LPS group, reaching the same levels exhibited by the control group. Propentofylline alone (PPF+SAL group) did not interfere with the depressive-like parameters, compared with the control group.

**Fig 5:**
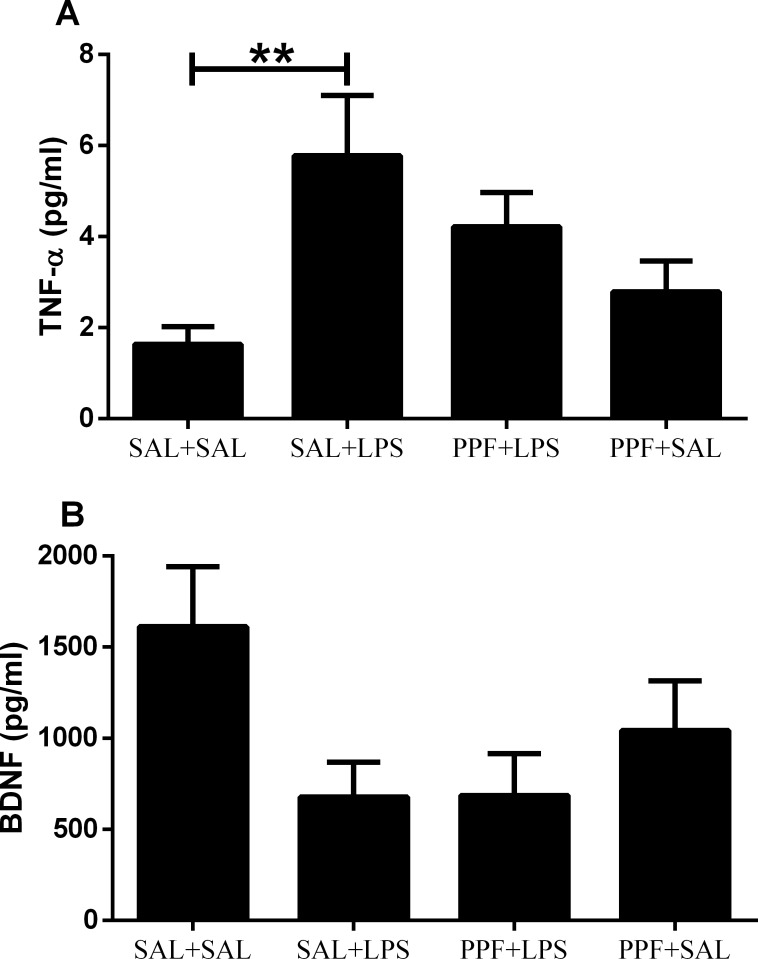
TNF-α and BDNF. Effects of LPS (1 mg/kg/day) and propentofylline (12.5 mg/kg/day) on TNF-α and BDNF plasma levels in adult male rats. SAL+SAL, saline injection at days 1–5 and another saline injection 1 h later at days 3–4; SAL+LPS, saline injection at days 1–5 and LPS injection 1 h later at days 3–4; PPF+LPS, propentofylline injection at days 1–5 and LPS injection 1 h later at days 3–4; PPF+SAL, propentofylline injection at days 1–5 and saline injection 1 h later at days 3–4 (*n* = 10 per group). **p < 0.01 (one-way ANOVA followed by the Newman-Keuls test). The data are expressed as the mean ± SEM.

The plasmatic TNF-α levels were different between groups (S2 Table). LPS (SAL+LPS group) increased the TNF-α levels, compared with the control group (Fig 5). Propentofylline treatment together with LPS (PPF+LPS group) resulted in similar TNF-α levels as those of control group, thus, preventing the TNF-α increase induced by LPS. Propentofylline alone (PPF+SAL group) did not interfere with the plasmatic TNF-α levels, compared with the control group. The plasmatic BDNF levels did not vary significantly among the four groups (Fig 5).

**Fig 6:**
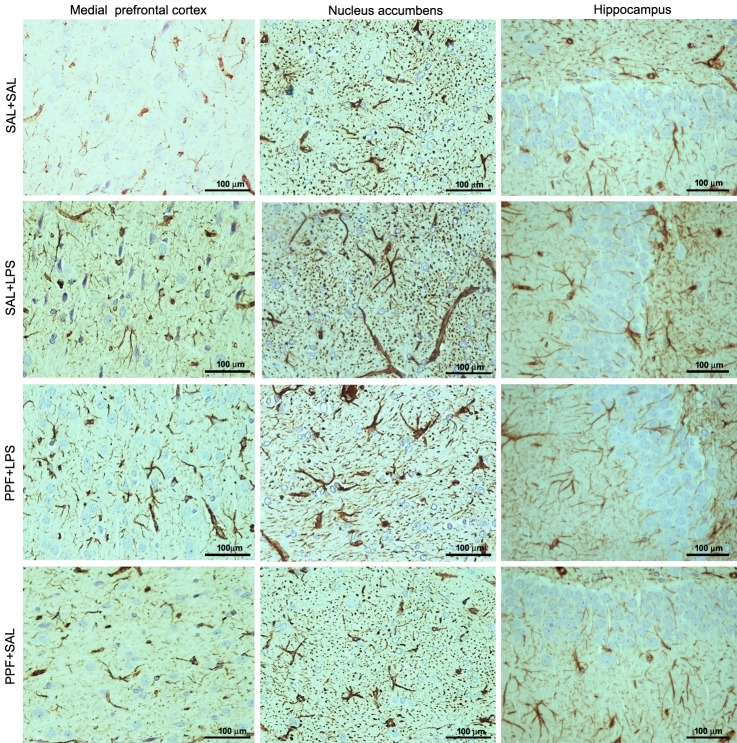
GFAP photomicrographs. Effects of LPS (1 mg/kg/day) and propentofylline (12.5 mg/kg/day) on central glial fibrillary acidic protein (GFAP) expression. Photomicrographs of the medial prefrontal cortex, nucleus accumbens, and hippocampus analyzed by immunohistochemistry in adult male rats. SAL+SAL, saline injection at days 1–5 and another saline injection 1 h later at days 3–4; SAL+LPS, saline injection at days 1–5 and LPS injection 1 h later at days 3–4; PPF+LPS, propentofylline injection at days 1–5 and LPS injection 1 h later at days 3–4; PPF+SAL, propentofylline injection at days 1–5 and saline injection 1 h later at days 3–4.

**Fig 7:**
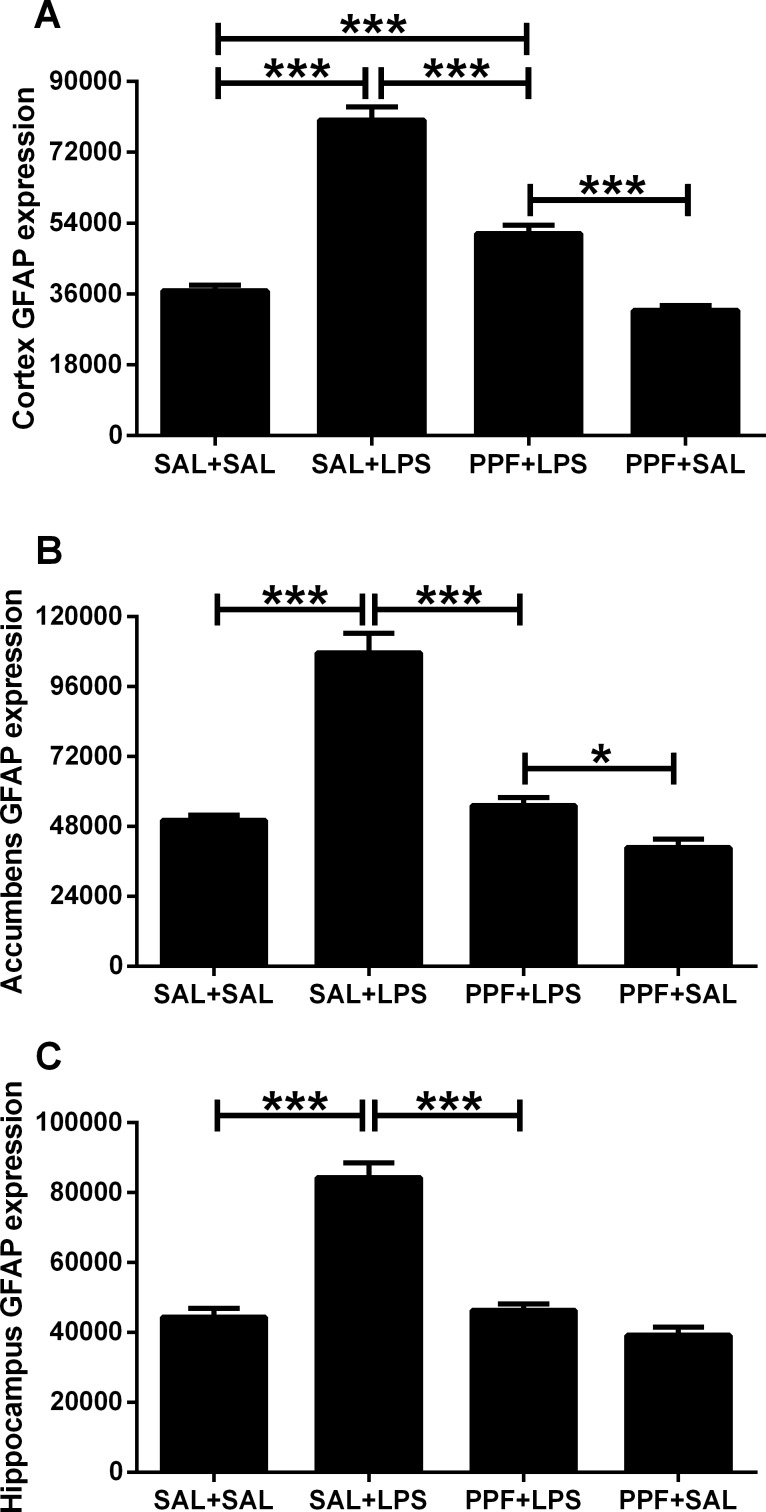
GFAP expression. Effects of LPS (1 mg/kg/day) and propentofylline (12.5 mg/kg/day) on glial fibrillary acidic protein (GFAP) expression in the medial prefrontal cortex, nucleus accumbens, and hippocampus, analyzed by immunohistochemistry in adult male rats. SAL+SAL, saline injection at days 1–5 and another saline injection 1 h later at days 3–4; SAL+LPS, saline injection at days 1–5 and LPS injection 1 h later at days 3–4; PPF+LPS, propentofylline injection at days 1–5 and LPS injection 1 h later at days 3–4; PPF+SAL, propentofylline injection at days 1–5 and saline injection 1 h later at days 3–4 (*n* = 10 per group). *p < 0.05 and ***p < 0.0001 (one-way ANOVA followed by the Newman-Keuls test). The data are expressed as the mean ± SEM.

The medial prefrontal cortex, nucleus accumbens, and hippocampus GFAP expressions were different between groups (S2 Table). LPS (SAL+LPS group) increased the GFAP expression in these brain areas, compared with the control group (Figs 6 and 7). Propentofylline treatment together with LPS (PPF+LPS group) prevented the increased GFAP expression induced by LPS in these brain areas, compared with SAL+LPS group. PPF+LPS reached the same levels exhibited by the control group in the nucleus accumbens and hippocampus, but not in medial prefrontal cortex. Propentofylline alone (PPF+SAL group) did not interfere with the GFAP expression in these brain areas, compared with the control group.

## Discussion

LPS induced body weight loss, open field behavior impairments (decreased locomotion and rearing frequencies, and increased immobility time), and increased plasmatic TNF-α levels in rats, compared with control group. Thus, LPS induced sickness behavior 24 and 48 hours after initial exposure. Repetitive LPS administration also increased the immobility time and reduced the climbing time in the forced swim test, compared with the control group, i.e., LPS induced depressive-like behavior in rats. LPS inducing sickness behavior and depressive-like behavior was expected [24, 26, 34, 35], and shows that the model applied in the present study was adequate.

We induced sickness behavior and depressive-like behavior to study the potential beneficial effect of propentofylline to treat them. Propentofylline prevented body weight loss and open field behavior impairments induced by LPS after four days of consecutive treatment. Thus, propentofylline prevented the sickness behavior. Similarly, propentofylline prevented the impairments found in the forced swim test induced by LPS after five days of consecutive propentofylline treatment, i.e., it prevented the depressive-like behavior. In addition to the statistical difference between SAL+LPS and PPF+LPS groups, it is important to mention that all analyzed parameters, including TNF-α levels, presented no statistical differences when comparing control (SAL+SAL) and PPF+LPS groups, corroborating the beneficial effect of propentofylline to treat sickness behavior and/or depressive-like behavior.

It was expected LPS inducing both sickness behavior and depressive-like behavior, because several studies from different groups have already shown this scenario [26, 27, 34, 35]. There are studies with different doses, other species, and other behavioral and molecular tests revealing similar results [24, 26, 27, 44–47]. This shows that the effect of LPS inducing sickness behavior and depressive-like behavior is quite preserved. However, at the day rats were evaluated in the forced swim test, those that received LPS still had impairments in locomotor activity in the open-field. This long-lasting effect may have influenced the performance of the animals in the light-dark and forced swim tests, two behavioral assessments with a considerable locomotor component. Usually, studies refer this effect as “depressed locomotor activity” [45, 46]. Thus, it is difficult to dissociate sickness behavior from depressive-like behavior in the present study. This result should also be taken into account when extrapolating the antidepressant activity of propentofylline.

We also studied the anxiety-like behavior in the light-dark test. All the anxiety-related parameters, i.e., dark side entry latency, total time spent in the dark side, and total time spent in the light side did not show statistical differences between LPS and control groups. Therefore, LPS did not interfere with anxiety levels. This result is in accordance with our previous study with acute LPS administration in rats [48]. Observing other behavioral parameters related to anxiety (time spent in central and peripheral zones in an open field) there were no differences between LPS and control groups. Presently, the only difference found in the light-dark test between LPS and control group was a decrease in rearing frequency, which is a motor/exploratory parameter (similar as in the open-field test), related to sickness behavior [34]. Thus, LPS did not influence anxiety-like behavior, but motor/exploratory and motivational parameters.

Interestingly, although neither LPS nor propentofylline influenced the anxiety levels of rats, propentofylline treatment together with LPS decreased the time spent in the dark side, increasing the time spent in the light side of the light-dark apparatus, compared with the LPS group. Thus, compared with LPS group, but not with control group, propentofylline treatment together with LPS resulted in an anxiolytic effect.

In addition to proinflammatory cytokines, BDNF is being considered as a promising peripheral depressive biomarker [28, 29]. BDNF is a small protein found throughout the central nervous system, and peripheral blood. It regulates neuronal survival, morphology, development, and function and plays a critical role in synaptogenesis and synaptic plasticity [49]. BDNF appears to be involved in the genesis of many depression cases; several depressive patients present reduced BDNF levels [28, 29]. Moreover, a new class of antidepressant drugs related to BDNF interference expression has been studied [29, 50].

Presently, we did not find effects for LPS and propentofylline in plasmatic BDNF levels. Considering that both human and rat studies have demonstrated that BDNF levels in the blood reflect BDNF levels in the brain [51, 52], we concluded that sickness behavior and depressive-like behavior induced by repetitive LPS administration does not seem to be related with the BDNF pathway. Likewise, the beneficial effect of propentofylline to treat sickness behavior and depressive-like behavior was probably not a consequence of BDNF interferences.

The sickness behavior and depressive-like behavior found after repetitive LPS exposure were related to peripheral and central immune pro-inflammatory activation. We showed that LPS-treated rats presented elevated plasmatic TNF-α levels and GFAP expression in the medial prefrontal cortex, nucleus accumbens, and hippocampus. Astrocytes are dynamic cells that respond to changes in the central nervous system (CNS) by undergoing morphological and functional alterations that affect neuronal activity [53]. In response to CNS insults, astrocytes develop a hypertrophic or reactive phenotype termed astrogliosis [54], which is characterized by the upregulation of specific structural proteins, such as GFAP and vimentin [55]. Data in literature support the use of quantification of GFAP-immunolabelled areas in a predetermined area of CNS tissue as a sensitive and reliable method for showing the presence or absence of neuroinflammation to a wide range of injury stimuli [56, 57]. Thus, the present result of increased GFAP expression suggests a neuroinflammatory response after LPS exposure.

Moreover, peripheral and central immune markers presently studied revealed that the beneficial effect of propentofylline during sickness behavior and depressive-like behavior also happened through downregulation/attenuation of neuroinflammatory processes. TNF-α levels and GFAP increase in expression were prevented after propentofylline treatment even when rats received two high doses of LPS.

Even if propentofylline resulted in a beneficial effect during sickness behavior and/or depressive-like behavior, when propentofylline was administered alone, i.e., without an immune challenge, it resulted in some adverse effects. Consecutive propentofylline administration without LPS exposure increased the body weight and decreased exploratory behavior (rearing) in rats. This exploratory behavior decrease induced by propentofylline may be related to its direct action in the central dopaminergic system. Propentofylline has been reported to inhibit the release of dopamine during transient ischemia and modulate dopamine metabolism in the striatum in rats [58]. Moreover, prior administration of propentofylline dramatically abrogated the methamphetamine-induced dopamine peak effect [59]. They proposed that propentofylline may hamper the dopamine efflux through D2-autoinhibition. Thus, we would not suggest propentofylline administration to healthy subjects, but exclusively to those presenting sickness behavior and/or depressive-like behavior.

In conclusion, LPS administration induced sickness behavior and depressive-like behavior in rats via neuroinflammatory pathway. Propentofylline prevented both sickness behavior and depressive-like behavior, concerning behavioral and neuroimmune parameters. The present findings may contribute to a better understanding and treatment of depression and associated diseases.

## Supporting Information

S1 Table F and *p* of two-way analysis of variance. Statistical values of F and *p* of two-way analysis of variance of body weight and open field general activity.
(DOCX)

S2 Table F and *p* of one-way analysis of variance. Statistical values of F and *p* of one-way analysis of variance of light-dark test, forced-swim test, plasmatic evaluations, and astrocyte GFAP expression.
(DOCX)
