# Supplementary material for: Propentofylline Prevents Sickness Behavior and Depressive-Like Behavior Induced by Lipopolysaccharide in Rats via Neuroinflammatory Pathway
Source: PLoS One. 2017 Jan 5;12(1):e0169446. doi: 10.1371/journal.pone.0169446 (PMC5215944; doi:10.1371/journal.pone.0169446)
Supplement: S1 Table — Statistical values of F and p of two-way analysis of variance of body weight and open field general activity. (DOCX) [file pone.0169446.s001.docx]

**S1 Table. F and *p* of two-way analysis of variance.** Statistical values of F and *p* of two-way analysis of variance of body weight and open field general activity

|  | Body weight | Locomotion | Rearing | Immobility |
| --- | --- | --- | --- | --- |
| Interaction |  |  |  |  |
| F | 2.68 | 2.90 | 1.37 | 3.13 |
| *p* | 0.0024 ** | 0.0115 * | 0.2349 | 0.0073 ** |
| Treatment |  |  |  |  |
| F | 14.07 | 10.88 | 19.05 | 19.09 |
| *p* | < 0.0001 *** | < 0.0001 *** | < 0.0001 *** | < 0.0001 *** |
| Days |  |  |  |  |
| F | 6.06 | 13.69 | 19.97 | 19.96 |
| *p* | 0.0001 *** | < 0.0001 *** | < 0.0001 *** | < 0.0001 *** |

* p < 0.05; ** p < 0.01; *** p < 0.001.
