# Supplementary material for: Propentofylline Prevents Sickness Behavior and Depressive-Like Behavior Induced by Lipopolysaccharide in Rats via Neuroinflammatory Pathway
Source: PLoS One. 2017 Jan 5;12(1):e0169446. doi: 10.1371/journal.pone.0169446 (PMC5215944; doi:10.1371/journal.pone.0169446)
Supplement: S2 Table — Statistical values of F and p of one-way analysis of variance of light-dark test, forced-swim test, plasmatic evaluations, and astrocyte GFAP expression. (DOCX) [file pone.0169446.s002.docx]

**S2 Table. F and *p* of one-way analysis of variance.** Statistical values of F and *p* of one-way analysis of variance of light-dark test, forced-swim test, plasmatic evaluations, and astrocyte GFAP expression

|  | F | *p* |
| --- | --- | --- |
| Light-dark test |  |  |
| Dark side entry latency | 2.06 | 0.1233 |
| Dark side total time | 5.68 | 0.0027 ** |
| Light side total time | 5.68 | 0.0027 ** |
| Rearing frequency | 5.20 | 0.0044 ** |
| Forced-swim test |  |  |
| First immobility latency | 0.44 | 0.7223 |
| Immobility time | 4.34 | 0.0104 * |
| Climbing time | 11.60 | < 0.0001 *** |
| Plasmatic evaluations |  |  |
| TNF-α | 4.37 | 0.0100 * |
| BDNF | 2.82 | 0.0524 |
| Astrocyte GFAP expression |  |  |
| Medial prefrontal cortex | 96.22 | < 0.0001 *** |
| Nucleus accumbens | 57.03 | < 0.0001 *** |
| Hippocampus | 50.09 | < 0.0001 *** |

* p < 0.05; ** p < 0.01; *** p < 0.001.
